# Psychological distress and musculoskeletal pain in manual therapists during the second wave of the COVID-19 pandemic in Sweden: a cross-sectional study

**DOI:** 10.1186/s12998-023-00511-2

**Published:** 2023-09-12

**Authors:** Nathan Weiss, Eva Skillgate, Iben Axén

**Affiliations:** 1 Unit of Intervention and Implementation Research for Worker Health, Institute of Environmental Medicine, Karolinska Institutet, 171 77 Stockholm, Sweden; 2 Department of Health Promotion Science, Musculoskeletal and Sports Injury Epidemiology Center, Sophiahemmet University, 114 86 Stockholm, Sweden; 3 Naprapathögskolan-Scandinavian College of Naprapathic Manual Medicine, 114 19 Stockholm, Sweden; 4 The Norwegian Chiropractic Research Foundation “Et liv I Bevegelse”, ELIB, Oslo, Norway

**Keywords:** Anxiety, Chiropractors, Depression, Naprapaths, SARS-CoV-2, Stress

## Abstract

**Background:**

The COVID-19 pandemic had an unprecedented impact on healthcare, and the health of healthcare workers has been subject of much research. However, studies of health-related factors in manual therapists during the COVID-19 pandemic are scarce. Research in this field can provide valuable insights for future crises policy and guidelines, including in regions where the public health response to COVID-19 contrasts with that of most other international jurisdictions. The aim was to describe the prevalence of psychological distress and musculoskeletal pain, and to investigate factors potentially associated with high psychological distress and activity-limiting musculoskeletal pain in clinically active chiropractors and naprapaths during the second wave of the COVID-19 pandemic in Sweden.

**Methods:**

A cross-sectional survey was distributed to a representative sample of Swedish manual therapists, between November 2020 and January 2021. High psychological distress and activity-limiting musculoskeletal pain were investigated regarding associations with residing in a municipality with a high spread of infection, a previous/ongoing SARS-CoV-2 infection, clinical interferences and economic consequences associated with the pandemic. Generalized Linear Models with log link and binomial distribution were used, computing prevalence ratios (PR) with 95% confidence intervals (95% CI).

**Results:**

A total of 762 participants were included, representing 46% of the source population. The prevalence of depressive, anxiety, and stress symptoms was 17%, 7%, and 12%, respectively. Neck (50%), low back (46%), upper back (40%), and shoulders (39%) were the most prevalent musculoskeletal pain areas. Economic consequences due to the pandemic were associated with high psychological distress (PR = 2.30, 95% CI: 1.48–3.53).

**Conclusions:**

During the second wave of the COVID-19 pandemic in Sweden, manual therapists primarily suffered from musculoskeletal pain related to the back and shoulders, while depressive symptoms were the most common symptom of psychological distress. Owners of businesses that suffered economic consequences had a higher prevalence of high psychological distress, which may call for targeted support of this group in future similar contexts. Future longitudinal studies during the pandemic are warranted to assess these associations further.

**Supplementary Information:**

The online version contains supplementary material available at 10.1186/s12998-023-00511-2.

## Introduction

A novel strain of virus, causing a local disease outbreak in Wuhan, Hubei province, China was identified at the end of 2019. It was identified as a coronavirus and termed SARS-CoV-2, with the resulting infectious disease termed Corona Virus Disease 19 (COVID-19) [1].

In January 2020, the first case of COVID-19 was confirmed in Sweden. Cases rose in many regions by April 2020, marking Sweden’s first COVID-19 wave. Subsequently, after transmission having stabilized during the summer, a second wave of increased cases and associated morbidity were recorded by the third quarter of 2020 [2]. As of December 2020, more than 460,000 cases of COVID-19 were confirmed in Sweden, including 10,000 deaths [3].

Unlike many other countries, Sweden’s COVID-19 strategy comprised no nationwide lockdowns and fewer and looser restrictions. The policy response included varying measures implemented over the course of the pandemic to mitigate the spread of COVID-19, and to alleviate the impact on health care capacity, individuals, and businesses. The core of Sweden’s response was characterized by non-binding restrictions emphasizing individual responsibility, including practicing social distancing, hand sanitizing, and staying home even with mild disease symptoms. Further restrictions and guidelines comprised working from home, switching schooling to remote lessons, avoiding unnecessary travel, and restricting public gatherings. The primary agency responsible for formulating Sweden’s COVID-19 guidelines and recommendations were The Public Health Agency of Sweden, additionally, regional guidelines were also composed to limit local spread, if necessary [2, 4].

Chiropractors and naprapaths are the two largest manual therapy professions in Sweden; treating, diagnosing, and rehabilitating common musculoskeletal pain disorders such as back- and neck pain and other conditions arising from the musculoskeletal system [5, 6]. Many manual therapists are private practitioners with sole ownership of their practice or owners in a group practice [7].

During the COVID-19 pandemic, manual therapists and their patients were potentially exposed to a more contagious work environment compared to that of many healthcare workers (HCWs) because the clinical encounter necessitates close physical contact between the practitioner and patient. Consequently, chiropractors and naprapaths are arguably at risk of contracting and infecting patients with SARS-CoV-2 in their clinical work. Direct contact transmission, i.e., person to person spread of SARS-CoV-2 through droplets or aerosols by infected individuals, especially in confined spaces such as in the healthcare setting is well documented [8, 9]. Other HCWs have previously expressed high levels of stress, worries, and anxiety of contracting infection transmitted from asymptomatic patients carrying SARS-CoV-2, or from patients having subclinical symptoms [10]. Occupational stress related to personal, and colleagues’ safety has also been reported among chiropractors amidst the pandemic [7]. The effects of psychosocial stressors related to the pandemic at large, such as limited interpersonal contact, isolation, fear, financial worry, ambiguous media, and news coverage as well as the inherent uncertainty regarding the pandemic’s duration may be associated with short- and long-term health consequences such as psychological distress and musculoskeletal pain [11, 12].

In a recent cross-sectional study conducted during the COVID-19 pandemic among chiropractors, 50% and 30% reported moderate and high levels of psychological distress, respectively [7]. In physiotherapists during the pandemic, 32% reported symptoms of anxiety, and 18.5% reported symptoms of depression [13]. Similarly, other studies investigating mental health aspects among physiotherapists during the pandemic reported a high prevalence of depressive, PTSD, and stress symptoms [14], as well as burnout [15].

To the best of our knowledge, no previous study has examined chiropractors’ and naprapaths’ psychological distress and musculoskeletal pain status during the COVID-19 pandemic in a Swedish context. Considering the potentially contagious work environment these manual therapists face in the context of the COVID-19 pandemic, the high burden of psychological distress found among other HCWs, studies examining health outcomes and factors associated with these are warranted. The aim of this study was to describe the prevalence of psychological distress and musculoskeletal pain, and to investigate factors associated with high psychological distress and activity-limiting musculoskeletal pain.

## Materials and methods

The study reports cross-sectional baseline data from the research project ‘Corona And Manual professions’ (CAMP); a national prospective cohort study with the overall aim to examine the impact of the COVID-19 pandemic on Swedish chiropractors’ and naprapaths’ health, work environment, and economy during a 12-month period. ClinicalTrials register identifier: NCT04834583.

### Eligibility criteria

Clinically active chiropractors and naprapaths, licensed by the National Board of Health and Welfare in Sweden or those undergoing licensing practice were eligible to participate in the study. Those with missing information regarding eligibility criteria and/or psychological distress and musculoskeletal pain were excluded.

### Recruitment

A register excerpt of licensed chiropractors and naprapaths in Sweden were obtained from the National Board of Health and Welfare in Sweden in October 2020 to identify eligible study participants [16]. Eligible study participants were informed and enrolled in the study through a comprehensive recruitment strategy: (I) information through professional associations’ internal channels, (II) information in profession-specific groups on social media, (III) information through larger clinics’ internal channels, (IV) up to three individual emails regarding the study, and (V) an information folder sent through ordinary mail. Study participants were included from November 2020 through January 2021, which was the time of the second wave of COVID-19 in Sweden. Study participants were enrolled through a generic link to information about the study and the baseline questionnaire (www.campstudy.se).

### Data collection

The data in the CAMP study were collected using four web-based surveys during a one-year period, each completed in 15–30 min. The current study is based on baseline data. Prior to data collection, non-standardized questions and items planned to be included in the baseline survey were reviewed in a focus group with a convenience sample of licensed naprapaths and naprapath students (n = 10), age range 23–26 with an equal gender distribution. The survey was sent in advance to focus group members who then were invited to participate in an audiotaped meeting; whereupon they were asked about the survey’s comprehensibility and potential improvements. The focus group discussions led to minor wording revisions of non-standardized questions. Moreover, the final version of the baseline survey was sent out to a small sample (n = 5) of eligible study participants approximately one week prior to recruitment to test its functionality. Data collection was managed using REDCap, an electronic data collection tool provided by Karolinska Institutet [17, 18]. The survey covered questions and instruments related to the domains: I) Demographic factors; II) Profession-related factors; III) Work environment; IV) Health-related factors; V) lifestyle factors; VI) Psychological factors; VII) Business’ economy. Detailed information regarding items and questions used in this study is presented in Additional file 1, Table 1. Participants with partial survey responses received an automated reminder up to three times if the survey was not completed. Furthermore, if no response was received on the fourth week upon first entering the study, a text message reminder was sent out, and subsequently participants was reminded by a phone call. Variables of interest for the present study are explained in detail below.

### Residing in a municipality with a high spread of infection

‘Residing in a municipality with a high spread of infection’ was operationalized as a proxy for practicing as a manual therapist in a geographical area with high rates of infectious spread, potentially leading to contracting SARS-CoV-2. The municipal spread of infection was measured and categorized as follows: study participants’ self-reported postal codes were matched with and categorized into Sweden’s 290 municipalities through data from PostNord Sweden [19]. Data on COVID-19 confirmed cases for 2020–2021 were acquired from The Public Health Agency of Sweden, stratified by municipality [3]. Data on the number of inhabitants in each Swedish municipality were obtained from Statistics Sweden [20]. Furthermore, the cumulative incidence of confirmed cases per 100,000 inhabitants each week of enrollment was calculated for each municipality. The cumulative incidence for each participant’s municipality of residence the preceding four-week period upon enrollment was considered. The municipalities with a rate of confirmed cases in the 50^th^ percentile was classified as municipalities with a high spread of infection for the respective four-week period.

### Previous or ongoing SARS-CoV-2 infection

Classification of participants with previous or ongoing SARS-CoV-2 infection was done by asking participants whether they had contracted or currently had a SARS-CoV-2 infection with the response alternatives: “Yes (verified by test)”, “Probably”, “Probably not”, “No (verified by test), “Do not know”. Participants answering “Yes (verified by test)” or “Probably” were classified as having a previous/ongoing SARS-CoV-2 infection.

### Clinical interferences due to authorities’ recommendations

Participants were asked to comment on the following statement: “Authorities recommendations have interfered with clinical practice”, with the answer alternatives: “Not at all”, “Yes, to some degree”, “Yes, to a moderate degree”, “Yes, to a large degree”, and “I have not been clinically active during the COVID-19 pandemic”. Participants answering “Yes, to a moderate degree” or “Yes, to a large degree” were classified as having clinical interferences due to the pandemic.

### Economic consequences due to the pandemic

Business owners were asked about the expected revenue for 2020 compared to 2019. Those with an expected decrease greater than 25% were classified as having economic consequence due to the pandemic.

### Psychological distress

To assess participants’ psychological health, a previously validated and translated Swedish version of DASS-21 was used [21]. The DASS-21 questionnaire is a widely used self-reported measure of depressive, anxiety, and stress symptoms, used in both clinical and research settings with adequate psychometric properties, with the ability to differentiate between clinical and non-clinical cases [21–23]. The DASS-21 consists of 21 statements whereupon respondents rate how much a particular statement applied to them over the past 7-days, on a 4-point Likert scale, ranging from 0 “did not apply to me at all” to 3 “applied to me very much, or most of the time”; resulting in a total score between 0 and 63. Furthermore, the 21 statements are clustered into the three subgroups depression, anxiety, and stress, comprising 7 items each. A higher score on a particular subgroup represents a greater severity of symptoms [22, 23]. Depressive, anxiety, and stress symptoms were categorized according to the degree of severity: no/normal, mild, moderate, severe, and extremely severe through previously established cut-offs [23]. Participants with moderate, severe, or extremely severe depressive, anxiety, or stress symptoms were classified as having ‘high psychological distress’.

### Musculoskeletal pain

Musculoskeletal pain and activity-limiting pain were measured using a modified version of the Nordic Musculoskeletal Questionnaire (NMQ). NMQ is an extensively utilized screening questionnaire, designed to measure participants’ musculoskeletal problems in epidemiological studies, with satisfactory validity and reliability [24, 25]. Respondents were asked questions pertaining to eleven bodily locations: neck, jaw, shoulders, upper back, elbows, wrists/hands, low back, hips/thighs, knees, ankles/feet, and other bodily area. The area “other bodily area” was added in this modified version of the NMQ. Respondents were asked whether they had experienced any pain the preceding three-month period, for each of the eleven body areas (yes/no). Respondents reporting pain were asked to rate their average pain intensity on a Numeric Rating Scale (NRS) ranging from 0 (no pain) to 10 (worst imaginable pain) during the preceding three-month period, for that particular body area. Lastly, irrespective of pain status, respondents answered whether they had been limited in normal daily activities (such as work, studying, housekeeping) due to problems in each body location. Participants reporting activity-limiting pain in at least one of the eleven body areas described above during the preceding three-month period were classified as having ‘activity-limiting musculoskeletal pain’.

### Statistical analyses

Descriptive statistics were used to present demographic, health-, clinical-, and lifestyle-related factors for the study sample. Mean and standard deviation (SD) were calculated as measure of central tendencies and dispersion for normally distributed continuous variables whereas numbers and proportions were used for categorical variables.

Prevalence was calculated for musculoskeletal pain and activity-limiting pain for each body region, as well as psychological distress for the dimensions depression, anxiety, and stress according to the degree of severity. Moreover, median numeric pain intensity was calculated for each of the eleven body regions with the corresponding interquartile range (IQR).

To assess associations between residing in a municipality with a high spread of infection, a previous or ongoing SARS-CoV-2 infection, clinical interferences, and economic consequences due to the pandemic, and high psychological distress and activity-limiting musculoskeletal pain, generalized linear models (GLMs) with binomial distribution and log link were computed, adjusted for age and gender. Results from the GLMs are presented as prevalence ratios (PR) with corresponding 95% confidence interval (95% CI).

Age, gender, occupation, and geographic region were compared between participants included in the analyses, and eligible participants (total source population). Differences were analyzed with independent *t*-test for normally distributed continuous variables (age), and χ^2^ test for categorical variables (gender, occupation, and geographic region) to elucidate potential sampling bias in the study sample. Statistical analyses were executed in R version 4.1.2 [26].

## Results

### Participant characteristics

**Fig. 1:**
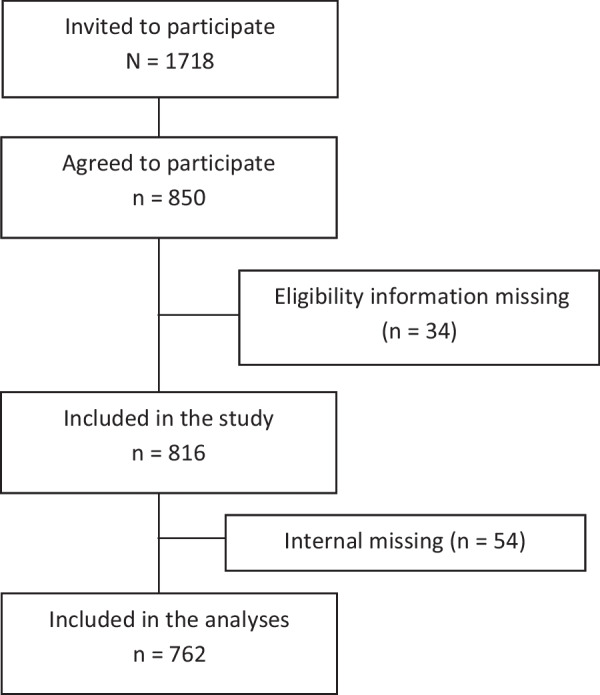
Study flow chart showing the number of invited participants, excluded participants, and included participants in the study and analyses

In the CAMP cohort, 1718 participants were invited, and 850 agreed to participate. Thirty-four participants were excluded due to not providing information regarding eligibility criteria, resulting in a study sample of 816, and a response proportion of 47%. After exclusion of 54 participants due to internal missing data on psychological distress and musculoskeletal pain, 762 were included in the final analyses (Fig. 1).

Characteristics of the study sample are presented in Table 1. The mean age of the sample was 44 years (SD =  ± 11.3), and 46% (n = 348) were women. The proportion of naprapaths and chiropractors amounted to 68% (n = 515) and 32% (n = 247), respectively. In total, 22% (n = 149) of the participants reported having contracted SARS-CoV-2. Furthermore, 20% (n = 152) and 43% (n = 329) reported a negative impact on their physical and psychological health due to the COVID-19 pandemic, respectively.

**Table 1:** Characteristics of study participants

Variable	*n*=^d^	Total
Age (years)^a^	762	44 ± 11.3
Gender	762	
Male^b^		414 (54%)
Female^b^		348 (46%)
Other^b^		0 (0%)
Occupation	762	
Licensed Naprapath^b^		470 (62%)
Licensed Chiropractor^b^		242 (32%)
Naprapath undergoing licensing practice^b^		45 (6%)
Chiropractor undergoing licensing practice^b^		5 (0%)
Number of hours clinically active/week previous 3 months^a^	760	28.4 ± 11.3
Other employment^b^	762	160 (21%)
Business owner	746	557 (75%)
Clinical interferences due to authorities’ recommendations	755	273 (36%)
Regular tobacco consumption	755	
Smoking^b^		3 (0%)
Snus^b^		119 (16%)
Number of days physically active/week^a^	755	3.8 ± 1.6
Self-reported previous/ongoing SARS-CoV-2 infection^b^	671	149 (22%)
Self-reported SARS-CoV-2 antibodies^b^	328	50 (15%)
Self-reported negative physical health due to COVID-19 pandemic^b^	762	152 (20%)
Self-reported negative psychological health due to COVID-19 pandemic^b^	762	329 (43%)
Medical condition^bc^	751	131 (17%)

^a^Values reported as mean and standard deviation (±)

^b^Values reported as number (n =) and percentage (%) of the sample

^c^Cancer, metabolic, cardiovascular, auto-immune, neurological disease

^d^Number of participants answering the question

Analyses comparing age, gender, occupation, and geographic region between the sample and eligible participants (total source population) showed that the sample constitutes 46% women, and the eligible 40% women (p = 0.01). There were no major differences observed regarding age, occupation, and geographic region [See additional file 1, Table 2].

**Table 2:** Prevalence of depressive, anxiety, and stress symptoms

Variable	No/normal	Mild	Moderate	Severe	Extremely severe
Depressive symptoms^a^	625 (83%)	63 (8%)	56 (7%)	12 (2%)	3 (0%)
Anxiety symptoms^a^	708 (93%)	20 (3%)	24 (3%)	0 (0%)	7 (1%)
Stress symptoms^a^	670 (88%)	48 (6%)	24 (3%)	13 (2%)	4 (1%)

^a^Values reported as number (n =) and percentage (%) of the sample

### Prevalence of psychological distress and musculoskeletal pain

The prevalence of depressive, anxiety, and stress symptoms of any severity degree was 17% (n = 131), 7% (n = 51), and 12% (n = 89), respectively. Furthermore, the prevalence of moderate or higher degrees of depressive symptoms was 9% (n = 71), anxiety symptoms 4% (n = 31), and stress symptoms 6% (n = 41) (Table 2).

The prevalence of musculoskeletal pain, activity-limiting pain, and median pain intensity for each of the eleven body areas are presented in Table 3. In total, 83% (n = 632) of the participants reported pain in at least one body area during the past three months. Pain was most prevalent in the neck, low back, upper back, and shoulders. Low back and wrists/hands were the most frequently reported activity-limiting locations (Table 3).

**Table 3:** Prevalence of musculoskeletal pain, pain intensity, and activity-limiting pain

Body region	*n*=^d^	Prevalence^a^	*n*=^d^	Pain intensity^b^	*n*=^d^	Activity-limiting pain^a^
Neck	762	384 (50%)	384	3.0 (2.0)	762	51 (7%)
Jaw	762	101 (13%)	100	3.0 (2.3)	762	8 (1%)
Shoulders	762	293 (39%)	290	4.0 (2.0)	761	57 (8%)
Upper back	762	303 (40%)	303	3.0 (2.0)	762	24 (3%)
Low back	762	353 (46%)	351	4.0 (2.0)	762	81 (11%)
Elbows	762	108 (14%)	106	3.0 (3.0)	762	27 (4%)
Wrists/hands	762	234 (30%)	233	4.0 (2.0)	762	71 (9%)
Hips/thighs	762	150 (20%)	149	4.0 (2.0)	762	37 (5%)
Knees	762	176 (23%)	175	4.0 (3.0)	761	51 (7%)
Ankles/feet	762	123 (16%)	122	4.0 (3.0)	762	41 (5%)
Other region^c^	762	45 (6%)	44	5.0 (4.0)	762	19 (3%)

^a^Values reported as number (n =) and percentage (%) of the sample

^b^Values reported as median and interquartile range

^c^Other region comprised head, groin, chest, calves, forearms, pelvis

^d^Number of participants answering the question

### Associations between characteristics and the prevalence of high psychological distress and activity-limiting musculoskeletal pain

Among the study participants, a total of 96 individuals were classified as having high psychological distress. Economic consequences due to the pandemic among business owners were associated with high psychological distress PR = 2.30 (95% CI: 1.48–3.53). Participants residing in a municipality with a high spread of infection the preceding four-week period upon enrollment had a PR of 1.40 (95% CI: 0.96–2.06) of having high psychological distress. Furthermore, the PR for clinical interferences due to authorities’ recommendations was 1.37 (95% CI: 0.93–1.98) of high psychological distress (Table 4).

**Table 4:** Prevalence and associations between characteristics and high psychological distress and activity-limiting musculoskeletal pain

Variable	High psychological distress			Activity-limiting musculoskeletal pain		
	*n* =	Prevalence (*n*, *%*)	PR (95% CI)	*n* =	Prevalence (*n*, *%*)	PR (95% CI)
Total	759	96 (13)		762	265 (35)	
Residing in a municipality with a high spread of SARS-CoV-2 infection^a^						
Yes	375	56 (7)	1.40 (0.96–2.06)	357	113 (15)	0.84 (0.69–1.02)
No	384	40 (5)	1.0	383	145 (20)	1.0
Previous/ongoing SARS-CoV-2 infection^a^						
Yes	149	16 (2)	0.86 (0.50–1.40)	149	61 (9)	1.23 (0.97–1.53)
No	519	66 (10)	1.0	522	176 (26)	1.0
Clinical interferences due to authorities’ recommendations^a^						
Yes	273	42 (6)	1.37 (0.93–1.98)	273	106 (14)	1.20 (0.98–1.47)
No	479	53 (7)	1.0	482	157 (21)	1.0
Economic consequences due to the pandemic^a^						
Yes	119	27 (5)	2.30 (1.48–3.53)	119	50 (10)	1.29 (0.99–1.65)
No	401	40 (8)	1.0	401	130 (25)	1.0

*CI* Confidence Interval, *PR* prevalence ratio

^a^Adjusted for age and gender

A total of 265 participants reported activity-limiting musculoskeletal pain in at least one body area the preceding three months upon entering the study. Participants with a previous/ongoing SARS-CoV-2 infection, clinical interferences due to authorities’ recommendations, and economic consequences due to the pandemic had a PR of activity-limiting musculoskeletal pain between 1.20 and 1.29, albeit not statistically significant, see Table 4.

## Discussion

To the best of our knowledge, this is the first study to evaluate chiropractors’ and naprapaths’ psychological distress and musculoskeletal pain during the COVID-19 pandemic in Sweden, and factors associated with these.

### Prevalence of psychological distress

In the present study, symptoms of depression, stress, and anxiety were reported by 17%, 12%, and 7% of the participants, respectively. The pre-pandemic point prevalence of severe depressive and mild to moderate anxiety symptoms among the Swedish general population were 4–11%, and 15–39%, respectively [27, 28]. Furthermore, data from The Public Health Authority in Sweden indicates that these figures have not changed substantially during the COVID-19 pandemic in Sweden [29]. Thus, it seems that our sample reported lower prevalence of depression and anxiety symptoms than the general population at this time. Compared to HCWs during the pandemic, the prevalence of depressive, anxiety, and stress symptoms were also lower in our study. Among chiropractors during the early phases of the pandemic in the US, 51%, and 30% reported moderate, and high stress levels, respectively [7]. Likewise, in a sample of physiotherapists in South Korea, 32% reported depressive symptoms, and 19% reported anxiety symptoms [13]. Similar figures have been documented in diverse groups of HCWs amidst the pandemic, in different contexts and countries [30–32]. However, as the pre-pandemic prevalence of psychological distress in our study population is not known, direct comparisons cannot be made.

Manual therapists that were owners of businesses with economic consequences due to the pandemic had a significant higher prevalence of high psychological distress in our study compared to owners of businesses without economic consequences. Financial stress was also reported to be one of the largest contributors to psychological distress among US chiropractors during the early phases of the pandemic [7]. We found that participants residing in a municipality with a high spread of SARS-CoV-2 infection had higher psychological distress than participants residing in municipalities with low spread. This finding is in line with a study conducted during the early phases of the COVID-19 pandemic in China [33], where front-line HCWs in the Hubei province, i.e., the epicenter of the pandemic, had higher proportions of anxiety and stress symptoms compared to other HCWs in other provinces in China. Similarly, the case count of COVID-19 was associated with longitudinal perceived stress in HCWs in New York City [34].

### Prevalence of musculoskeletal pain

Our findings revealed that pain in the neck (50%), low back (46%), upper back (40%), and shoulders (39%) were the most prevalent reported areas in our sample. Overall, our sample consistently had a higher prevalence of musculoskeletal pain compared to other studies’ estimates during and before the COVID-19 pandemic, measured with the NMQ. In a large pre-pandemic general population sample in Australia, the 12-month prevalence of musculoskeletal pain was 78% [35], compared to a three-month prevalence of 83% in our study. Similarly, in a general population sample in Turkey, a prevalence of neck (19%), upper back (18%), and shoulders (14%) was reported during the pandemic [36]. However, our results are somewhat in line with a general population sample in Saudi Arabia after a three-month lockdown during the pandemic, where pain in the low back (44%), neck (33%), shoulders (23%), and upper back (16%) were most prevalent [37].

Activity-limiting musculoskeletal pain was more prevalent among manual therapists that were business owners with economic consequences due to the pandemic in our study. Economic hardship during crises has, in a previous study, been associated with the incidence of musculoskeletal pain [38]. Participants with a previous or ongoing SARS-CoV-2 infection had a higher prevalence of activity-limiting musculoskeletal pain compared to those who were not infected. This finding is in line with previous studies evaluating the acute and long-term effects of SARS-CoV-2 infection. In a systematic review conducted by da Rosa Mesquita et al., myalgia was one of the most frequent symptoms of a SARS-CoV-2 infection [39]. Furthermore, musculoskeletal pain has also frequently been reported by individuals suffering from post-acute covid syndrome [40].

However, the pre-pandemic prevalence of musculoskeletal symptoms in our sample is not known.

### Methodological considerations

A major methodological strength is the large sample size of our study (n = 762) which constitutes 44% of the total eligible source population. Furthermore, comparisons with eligible participants regarding age, gender, occupation, and geographical distribution only showed differences with regards to gender where a larger proportion of the sample were women (46% versus 40%), which indicates a representative sample, and a low risk of sampling bias. Consequently, this strengthens the study’s external validity to extrapolate the findings to clinically active chiropractors and naprapaths during the COVID-19 pandemic in Sweden. Symptoms of psychological distress and musculoskeletal pain were measured using standardized questionnaires with adequate psychometric properties, which lessens the risk of misclassification of the prevalence measures in the study. The definition of economic consequence (expected decrease of 25% or more) was deemed impactful by the research team but was not verified against other studies. Thus, the association between psychological distress and economic consequence could be both over- and under-estimated. Prior to conducting the analyses, ECDC’s categorization of color-coding countries ‘dark red’ during the time of data collection, i.e., 14-day cumulative cases ≥ 150 per 100.000 inhabitants were intended to be used as a cut-off for ‘residing in a municipality with a high spread of SARS-CoV-2’ [41, 42]. However, most of the Swedish municipalities had confirmed cases above this cut-off, which resulted in few unexposed participants with this categorization. Therefore, we used the arbitrary cut-off of median split, as this is a commonly utilized method of categorizing variables. However, arbitrary cut-offs have previously been shown to have less precision and statistical power, and underestimate observed relations compared to a continuous measure [43]. Non-standardized questions and items used in the survey were reviewed in a focus group meeting prior to the initiation of the data collection, to ensure comprehensibility of the questionnaire. However, the wording of the question regarding previous/ongoing SARS-CoV-2 infection limited the ability to differentiate between ongoing of previous infections. The cross-sectional design enabled prevalence measures, as well as associations between multiple variables. However, as all variables were measured at the same time-point, no causal association could be investigated based on the baseline results of the CAMP cohort.

## Conclusions

Depressive symptoms were the most prevalent psychological distress symptom reported among manual therapists during the second wave of the COVID-19 pandemic in Sweden, and pain was most prevalent in the neck, low back, upper back, and shoulders. Economic consequences due to the pandemic among business owners were associated with high psychological distress. These results will improve the understanding of the impact on COVID-19 on manual health care professions.

### Supplementary Information

**Additional file 1**: **Table S1**: Description of the variables included; **Table S2**. Characteristics of participants included in the analyses and eligible participants (source population) and results from χ2 test and independent samples t-test presented as p-values.

## Data Availability

The datasets generated and/or analyzed during the current study are not publicly available due to ethical considerations and the general data protection regulation (GDPR), but are available from the corresponding author on reasonable request.

## Abbreviations

COVID-19: Corona Virus Disease 2019
SARS-CoV-2: Severe Acute Respiratory Syndrome Coronavirus 2
HCWs: Healthcare workers
PTSD: Post Traumatic Stress Disorder
CAMP: Corona And Manual Professions
DASS-21: Depression Anxiety Stress Scale 21
NMQ: Nordic Musculoskeletal Questionnaire
SD: Standard Deviation
IQR: Interquartile Range
GLM: Generalized Linear Model
PR: Prevalence Ratio
95% CI: 95% Confidence Interval
US: The United States of America
