# Supplementary material for: Psychological distress and musculoskeletal pain in manual therapists during the second wave of the COVID-19 pandemic in Sweden: a cross-sectional study
Source: Chiropr Man Therap. 2023 Sep 12;31:34. doi: 10.1186/s12998-023-00511-2 (PMC10498599; doi:10.1186/s12998-023-00511-2)
Supplement: Supplementary file 1 — Additional file 1: Table S1: Description of the variables included; Table S2. Characteristics of participants included in the analyses and eligible participants (source population) and results from χ2 test and independent samples t-test presented as p-values. [file 12998_2023_511_MOESM1_ESM.docx]

This additional file describes detailed information regarding variables, included questions, and categorization in the analyses. Furthermore, demographic data comparing participants included in the analyses with eligible participants (total source population) are presented.

| **Table 1**. Description of the variables included. | | |
| --- | --- | --- |
| **Variable** | **Question^a^/questionnaire** | **Categorization** |
| Age | Age (number of years) | Continuous |
| Gender | Gender | Male/female/other |
| Occupation | Occupation | Licensed chiropractor/licensed naprapath/chiropractor during licensing practice/naprapath during licensing practice |
| Number of hours clinically active/week previous 3 months | ‘Average number of hours clinically active/week during the last three months’ | Continuous |
| Other employment | ‘I have another occupation’ | Yes/no |
| Business owner | ‘Do you have your own business within chiropractic/naprapathy?’ | Yes/no |
| Clinical interferences due to authorities’ recommendations | ‘Authorities’ recommendations have interfered with clinical practice’   - Not at all - Yes, to some degree - Yes, to a moderate degree - Yes, to a large degree - I have not been clinically active during the COVID-19 pandemic | Participants answering “Yes, to a moderate degree” or “Yes, to a large degree” were classified as having clinical interferences due to the pandemic |
| Regular tobacco consumption | ‘Do you smoke daily?’  ‘Do you use snus daily?’ | Yes/no |
| Number of days physically active/week | ‘How many days per week do you exercise?’   - (0-7) | Continuous |
| **Table 1.** (Continued). | | |
| Self-reported previous/ongoing SARS-CoV-2 infection | ‘Do you have/have you had COVID-19?’   - Yes (verified by test) - Probably - Probably not - No (verified by test) - Do not know | Participants answering “Yes (verified by test)” or “Probably” were classified as having a previous/ongoing SARS-CoV-2 infection |
| Self-reported negative physical health due to the COVID-19 pandemic | ‘Has your physical health been impacted by the COVID-19 pandemic?’   - Yes, very negative - Yes, negative - No, not at all - Yes, positive - Yes, very positive | Participants answering “Yes, very negative, or “Yes, negative” were classified as having negative physical health due to the COVID-19 pandemic |
| Self-reported negative psychological health due to COVID-19 pandemic | ‘Has your psychological health been impacted by the COVID-19 pandemic?’   - Yes, very negative - Yes, negative - No, not at all - Yes, positive - Yes, very positive | Participants answering “Yes, very negative, or “Yes, negative” were classified as having negative psychological health due to the COVID-19 pandemic |
| Medical condition | Modified Comorbidity Questionnaire [1] | Currently diagnosed and suffering from any cancer, metabolic, cardiovascular, auto-immune, neurological disease |
| Psychological distress | Depression Anxiety Stress Scale-21 [2] | Described in detail in the article text |
| Musculoskeletal pain | Modified Nordic Musculoskeletal Questionnaire [3] | Described in detail in the article text |
| **Table 1**. (Continued). | | |
| Economic consequences due to the pandemic | (Only business owners answered the statement)  ‘Concerning the total revenue for 2020 (compared to 2019) you expect it to’   - Decrease with 10% or less - Decrease 11-25% - Decrease 26-50% - Decrease 51-75% - Decrease more than 75% - Same as 2019 - Increase with 10% or less - Increase 11-25% - Increase 26-50% - Increase 51-75% - Increase more than 75% - Can not estimate - Not relevant | Those with an expected decrease greater than 25% were classified as having economic consequence due to the pandemic |
| ^a^ Translated from Swedish. | | |

| **Table 2.** Characteristics of participants included in the analyses and eligible participants (source population) and results from χ^2^ test and independent samples *t*-test presented as p-values. | | | | | | |
| --- | --- | --- | --- | --- | --- | --- |
|  | **Included in the analyses** | |  | **Eligible participants** | |  |
| **Variable** | ***n=*** |  |  | ***N=*** |  | **p-value** |
| Total | 762 |  |  | 1718 |  |  |
| Gender^a^ | 762 |  |  | 1718 |  | **.01*** |
| Male |  | 414 (54%) |  |  | 1024 (60%) |  |
| Female |  | 348 (46%) |  |  | 694 (40%) |  |
| Age (years)^b^ | 762 | 44 ±11.3 |  | 1667 | 44 ±10.8 | .82 |
| Occupation^a^ |  |  |  |  |  | .08 |
| Naprapath |  | 515 (68%) |  |  | 955 (64%) |  |
| Chiropractor |  | 247 (32%) |  |  | 377 (36%) |  |
| Geographical location (region)^a^ | 762 |  |  | 1683 |  | .77 |
| Blekinge |  | 8 (1%) |  |  | 13 (1%) |  |
| Dalarna |  | 16 (2%) |  |  | 37 (2%) |  |
| Gotland |  | 9 (1%) |  |  | 12 (1%) |  |
| Gävleborg |  | 10 (1%) |  |  | 47 (3%) |  |
| Halland |  | 15 (2%) |  |  | 34 (2%) |  |
| Jämtland |  | 9 (1%) |  |  | 26 (1%) |  |
| Jönköping |  | 19 (2%) |  |  | 30 (2%) |  |
| Kalmar |  | 11 (1%) |  |  | 22 (1%) |  |
| Kronoberg |  | 6 (0%) |  |  | 14 (1%) |  |
| Norrbotten |  | 10 (1%) |  |  | 17 (1%) |  |
| Skåne |  | 78 (10%) |  |  | 153 (9%) |  |
| Stockholm |  | 346 (48%) |  |  | 800 (48%) |  |
| Södermanland |  | 22 (3%) |  |  | 44 (3%) |  |
| Uppsala |  | 28 (4%) |  |  | 68 (4%) |  |
| Värmland |  | 16 (2%) |  |  | 25 (1%) |  |
| Västerbotten |  | 17 (2%) |  |  | 32 (2%) |  |
| Västermanland |  | 13 (2%) |  |  | 31 (2%) |  |
| Västernorrland |  | 13 (2%) |  |  | 44 (3%) |  |
| Västra Götaland |  | 71 (9%) |  |  | 151 (9%) |  |
| Örebro |  | 21 (3%) |  |  | 40 (2%) |  |
| Östergötland |  | 24 (3%) |  |  | 43 (2%) |  |
| ^a^ Values reported as number (n =) and percentage (%).  ^b^ Values reported as mean and standard deviation (±).  * Statistical significance (p = ≤ 0.05). | | | | | | |

**References**

1. Sangha, O.; Stucki, G.; Liang, M.H.; Fossel, A.H.; Katz, J.N. The Self-Administered Comorbidity Questionnaire: a new method to assess comorbidity for clinical and health services research. *Arthritis Rheum.* **2003**, *49*, 156-163, doi:10.1002/art.10993.

2. Kuorinka, I.; Jonsson, B.; Kilbom, A.; Vinterberg, H.; Biering-Sørensen, F.; Andersson, G.; Jørgensen, K. Standardised Nordic questionnaires for the analysis of musculoskeletal symptoms. *Appl. Ergon.* **1987**, *18*, 233-237, doi:10.1016/0003-6870(87)90010-x.

3. Alfonsson, S.; Wallin, E.; Maathz, P. Factor structure and validity of the Depression, Anxiety and Stress Scale-21 in Swedish translation. *J. Psychiatr. Ment. Health Nurs.* **2017**, *24*, 154-162, doi:https://doi.org/10.1111/jpm.12363.
